# Single-stage nonintubated uniportal thoracoscopic resection of synchronous bilateral pulmonary nodules after coil labeling: A case report and literature review

**DOI:** 10.1097/MD.0000000000006453

**Published:** 2017-03-24

**Authors:** Miao Zhang, Tao Wang, You-Wei Zhang, Wen-Bin Wu, Heng Wang, Rong-Hua Xu

**Affiliations:** a Department of Thoracic Surgery; b Department of Imaging; c Department of Medical Oncology; d Department of Orthopedics, Xuzhou Central Hospital Affiliated to Southeast University, Xuzhou, China.

**Keywords:** carbohydrate antigen (CA), coil labeling, computed tomography (CT), ground-glass nodule (GGN), ground-glass opacity (GGO), nonintubated, video-assisted thoracic surgery (VATS)

## Abstract

**Rationale:**

Preoperative localization of small pulmonary nodules is essential for precise resection, besides, the optimal treatment for pulmonary nodules is controversial and the prognosis without surgery is uncertain.

**Patient concerns:**

Herein we present a patient with compromised pulmonary function harboring synchronous triple ground-glass nodules located separately in different pulmonary lobes.

**Diagnoses:**

The pathological diagnosis of the nodules were chronic inflammation, inflammatory pseudotumor and atypical adenomatous hyperplasia, respectively.

**Interventions:**

The patient underwent single-stage, non-intubated thoracoscopic pulmonary wedge resection after computed tomography-guided coil labeling of the nodules.

**Outcomes:**

The postoperative recovery was encouragingly fast without obvious complications.

**Lessons:**

Non-intubated thoracoscopic pulmonary wedge resection is feasible for patients with compromised lung function, meanwhile, preoperative coil labeling of small nodules is reliable.

## 1 Introduction

Pulmonary ground-glass nodules (GGNs) are a common incidental finding on computed tomography (CT),^[1]^ followed by difficulties in diagnosis (benign, malignant, or metastatic tumors) and treatment (surgery or ongoing follow-up). The differentiation of synchronous primary tumors and intrapulmonary metastasis could be difficult, and minimally invasive surgery might be a proper choice for this dilemma.

It is reported that 9.5% of the pulmonary nodules are at a low risk, 79.6% are at a moderate risk, and 10.8% are at a high risk of malignancy, and a substantial fraction of intermediate-sized nodules turn out to be lung cancer ultimately.^[2]^ Curative intent shows benefits for patients with synchronous multiple primary lung cancers, as timely resection allows accurate diagnosis.^[3]^ Therefore, if resection can be performed, an aggressive approach is often warranted,^[4]^ and single-stage bilateral resection of synchronous multiple nodules is reported to be feasible and safe.^[5]^

Moreover, preoperative tissue diagnosis does not significantly influence the clinical outcomes of patients with persistent malignant-looking GGNs after surgical resection.^[6]^ Furthermore, precise resection of deeply situated small pulmonary nodules (<15 mm) is challenging. CT-guided percutaneous biopsy of small nodules is often difficult, along with the risks of pneumothorax and hemoptysis. In our institute, localization of small nodules by CT-guided hookwire insertion before surgery is replaced by coil labeling, because the hookwire is not reliable for all cases.

Herein, a patient with synchronous triple pulmonary nodules located in different lobes is presented for discussion, as he underwent single-stage resection of the nodules assisted with coil labeling.

## 2 Case presentation

**Figure 1:**
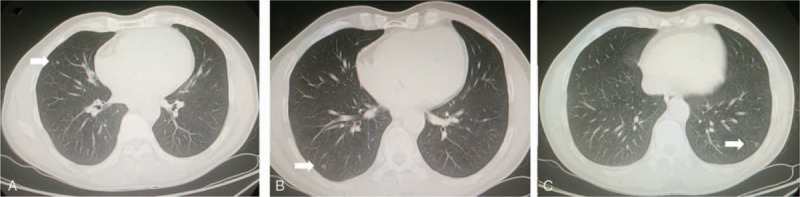
The CT on admission revealed synchronous triple pulmonary nodule measuring 5 to 7 mm in diameter located separately in different lobes.

A 57-year-old male patient was admitted to thoracic surgery department on April 17, 2016 for asymptomatic, newly diagnosed bilateral synchronous pulmonary nodules during health examination. These nodules were located in the left lower lobe, the right middle and lower lobe measuring 5 to 7 mm in diameter, separately, without obviously enlarged mediastinal lymph nodes, as depicted by CT scan in Fig. 1.

The patient suffered from mild to moderate dyspnea and cough during the winter for nearly 10 years. He had a smoking history of at least 40 pack-years, meanwhile, he had been working as a coal miner for 7 years before admission. His family and social histories were unremarkable. Thorough physical examination failed to identify any other suspicious lesions. Routine tests for hepatic, renal, and coagulation function were normal. Hepatitis B and C, and human immunodeficiency virus tests were negative. In addition, the serum tumor markers including carcinoembryonic antigen, cytokeratin 19 fragment, squamous cell carcinoma, neuron-specific enolase, alpha fetal protein, and carbohydrate antigen (CA125, CA19-9) were all in normal range. Cardiopulmonary function tests indicated moderate compromised pulmonary function [forced expiratory volume (FEV_1_) = 1.8 L, FEV_1_% = 65%, and forced vital capacity (FVC) = 3.2 L]. The cranial magnetic resonance, abdominal CT, and bone emission computed tomography of the patient excluded other suspicious lesions. Besides, the score of Eastern Cooperative Oncology Group Performance Status of the patient was 1. Positron emission tomography as a reasonable choice for staging was not performed, because it was not covered by health insurance and expensive.

The newly emerged GGNs without calcification were just suspicious of malignancy. Nevertheless, if the GGNs enlarge and metastasize during observation, and turned out to be malignant, the patient would not tolerate radical lobectomy because of compromised pulmonary function. Therefore, resection and frozen pathology were recommended empirically to avoid delayed diagnosis. Fine-needle aspiration cytology before surgery was avoided because of local dissemination risk and small size of the nodules.

The safety of nonintubated procedure for thoracic surgery was demonstrated,^[7]^ therefore, single stage, nonintubated thoracoscopic surgery was decided for this case, which was approved by Ethics Committee of our hospital.

**Figure 2:**
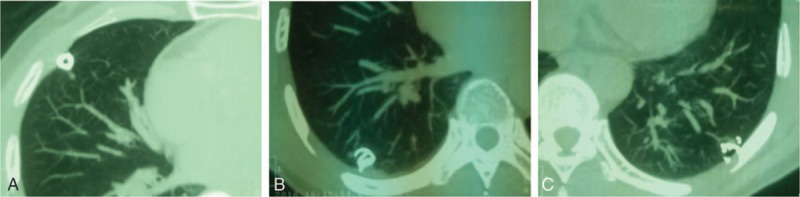
Pulmonary nodules of the patient were localized simultaneously by coil labeling, respectively.

Firstly, the bilateral triple GGNs in different lobes were simultaneously localized with CT-guided percutaneous insertion of embolization coil (Cook Medical, Bjaeverskov, Denmark), under local anesthesia, using 2% xylocaine (Recipharm Monts, Monts, France) before surgery (Fig. 2), followed by CT scan to confirm their correct location. The distance from the lesions to the visceral pleura surface was 10 to 20 mm, respectively. Then the patient was transported to the operating room.

Secondly, the anesthesia protocol for nonintubated surgery was underwent as reported, using a combination of target-controlled sedation, intercostal nerve block by 0.5% bupivacaine and intrathoracic vagal block.^[8,9]^ Nonintubated thoracoscopic wedge resection of the target pulmonary lobes was performed, assisted with carbon dioxide (CO_2_) artificial pneumothorax. The intrathoracic pressure was maintained at 10 to 12 mm Hg. In addition, devices for tracheal intubation and mechanical ventilation were also readily prepared for this patient.

The patient was positioned in the left lateral decubitus position for right-sided pulmonary wedge resection. An incision was made through the 5th intercostal space along the anterior axillary line. Rib spreading and retractor were avoided. A 10-mm video telescope was inserted for exploration of the pleural cavity. The incision was extended to 5 cm because of severe pleural adhesions, which was released carefully. The coils were easily found along the visceral pleura without dislocation or translocation. Lung tissue containing the nodule and the coil was lifted close to the incision by oval forceps to facilitate finger touch, and the nodules could be easily felt as protrusions. Then pulmonary wedge resection and mediastinal lymph node sampling including 4th, 6th, 8th, and 9th stations were performed using endoscopic stapling and harmonic scalpel. Then a 24-French soft chest tube was placed through the incision. After that, the patient was turned to the right lateral decubitus position for wedge resection of the left lower lobe.

**Figure 3:**
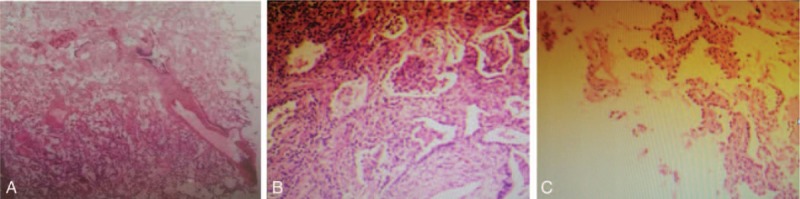
(A and B) Pulmonary nodules located in the right middle and lower lobes were diagnosed as inflammation. (C) Pulmonary GGN located in the left lower lobe was diagnosed as atypical adenomatous hyperplasia, by H-E staining (×100).

The total operation for triple pulmonary wedge resections lasted for 150 minutes, with intraoperative blood loss about 200 mL. Besides, the hemodynamic index including PaO_2_, PaCO_2_, and central venous pressure were stable throughout the operation. The nodules in right middle and right lower lobe were turned out to be chronic pulmonary inflammation (Fig. 3), while the nodule in the left lower lobe was confirmed pathologically as atypical adenomatous hyperplasia, with disease-free surgical margins. Furthermore, the sampled lymph nodes were tumor-negative.

The patient returned to normal activities 6 hours after surgery, without hemoptysis, dyspnea, arrhythmia, or vomiting, except for mild chest stuffiness and chest pain. The recovery of this patient was mainly uneventful, and he discharged 5 days after the operation. Subsequently, he was followed up remotely by internet-based approaches to deliver cardiopulmonary exercise education for 6 months up to now, who demonstrated satisfactory quality of life. Moreover, his pulmonary function was not significantly changed 3 months after the operation (FEV_1_ = 1.9 L, FEV_1_% = 67%, and FVC = 3.2 L).

## 3 Discussion

The optimal management for patients with multiple GGNs is controversial, and multidisciplinary and individualized should be considered. There are several issues concerning this dilemma need to be elucidated.

Firstly, the advent of CT screening for lung cancer would increase the incidence of pulmonary GGNs, but the screening is associated with a high frequency of false-positive results, because the high prevalent indeterminate are usually benign.^[10]^ GGNs with solid component, lobulation, spiculation, air cavity densities, pleural tags, vascular convergence sign, and a larger diameter on CT images are highly suggestive of malignancy.^[11]^ When pure GGNs are greater than 15 mm in diameter with nodularity, or have high pixel attenuation (>−472 HU), the nodules are more likely to be invasive adenocarcinomas.^[12]^ Besides, the risk factors of malignancy include nodule size>20 mm, age >60 years old, prior cancer history, current tobacco use, asbestos exposure, and speculated nodules.^[13]^ And invasive adenocarcinomas are diagnosed in approximately 1% of all pulmonary subsolid nodules.^[14]^ In addition, major resected GGNs are positive for epidermal growth factor receptor (EGFR), Kirsten rat sarcoma viral oncogene homolog (KRAS), anaplasticlymphoma kinase (ALK), or human epidermal growth factor receptor 2 (HER2) mutations, and EGFR mutation-positive tumors are correlated with minimally invasive adenocarcinoma or invasive adenocarcinoma and growth, compared with EGFR-negative tumors.^[15]^

Secondly, the natural course of persistent pulmonary GGNs with solid portions ≤5 mm differs significantly according to their nodule type and diameters.^[16]^ It is reported that the frequency of microscopic invasions, disease-free survival and recurrence between stage I pulmonary adenocarcinomas appearing as solid nodules and part-solid GGNs is similar.^[17]^ Additionally, selection of surveillance, biopsy, and resection for evaluation of pulmonary nodules can be complex, and patients’ preferences may not be consistently taken into account during the decision-making process, for absence of high-quality evidence regarding the optimal management.^[18]^ Therefore, a more systematic approach is necessary to ensure correct diagnosis and optimal management.^[19]^

Thirdly, the optimal therapy for GGNs remains controversial, because patients of this kind would take the risk of delayed diagnosis, for this reason, minimally invasive resection of the lesions might be reasonable to diminish risks of metastasis, however, the treatment depends on the cardiopulmonary reserve, and preference of the patients, comparing the benefits with potential harm of surgery. Surgical treatment is thought to be the most effective strategy for multiple GGNs, and single-stage bilateral resection in selected cases with synchronous bilateral multiple nodules is feasible.^[5]^ Nonintubated surgery is considered to minimize the side effects of double-lumen endotracheal intubation such as ventilation-induced lung injury and postoperative vomiting, with less hospital stay and decreased inflammatory cytokines, compared with endotracheal intubation under general anesthesia,^[7]^ and it is well tolerated by very elderly patients.^[20]^ In addition, thoracic epidural anesthesia in high-risk patients could avoid ventilator dependency, followed by fast recovery, which could be an alternative of conventional anesthesia for severely compromised patients,^[21]^ nevertheless, it is not suitable for patients with hemodynamic instability and severe pleural adhesions.

On the other hand, whether patients would benefit from limited resection is uncertain. The standard treatment of early lung cancer is minimally invasive lobectomy with systematic lymph node dissection, however, recent research has shown that some ground-glass opacity (GGO) lesions may be treated with sublobar resections.^[19]^ However, wedge resection should be carefully considered for patients with mixed GGO nodules because of the high recurrence rate,^[22]^ and segmentectomy is superior to wedge resection for tumors less than 20 mm in diameter.

Thirdly, there are several methods for localization of small nodules, including CT-guided patent blue vital dye injection, methylene blue dye injection, hookwire placement, coil labeling, lipiodol marking, and radiotracer placement. The methylene blue dye is not very reliable for its quick diffuse from the target site. Hookwire may cause tearing of the lung parenchyma with hemorrhage risk, and fail to localization if pneumothorax develops.^[23]^ CT-guided patent blue vital dye localization is more accurate, and it could be utilized for synchronous multiple nodules.^[8]^ It is hard to decide the proper margin of deeply located pulmonary nodules during the operation, thus, preoperative simulation using 3-dimensional CT and rapid prototyping of pulmonary vessels could be considered.^[24]^ The case presented herein shows satisfactory life quality during internet-based follow up, in accordance with the essential of fast track thoracic surgery.

It is noteworthy that indeterminate pulmonary nodules are commonly encountered and often result in costly and invasive procedures that eventually turn out to be unnecessary.^[25]^ Besides, invasive sampling of low-risk nodules and surgical resection of benign nodules remain common, suggesting a lack of adherence to guidelines for pulmonary nodules.^[2]^

## 4 Conclusion

In summary, the feasibility of single-stage nonintubated thoracoscopic pulmonary wedge resection for multiple coil-labeled GGNs is tentatively demonstrated from this case. However, more high-quality studies concerning standard of GGNs treatment and nonintubated thoracic surgery are required.
